# Quality improvement project to transition psychosocial oncology clinical care to a telehealth workflow during the COVID-19 pandemic: a quasi-experimental study

**DOI:** 10.1186/s12913-025-13609-5

**Published:** 2025-11-11

**Authors:** Rickinder Sethi, Brendan Lyver, Jaswanth Gorla, Robin Forbes, Kathleen A. Sheehan, Christian Schulz-Quach

**Affiliations:** 1 Department of Psychiatry, Faculty of Medicine, University of Toronto, Toronto, ON Canada; 2 Department of Supportive Care, Princess Margaret Cancer Centre, University Health Network, 200 Elizabeth St, Toronto, ON M5G 2C4 Canada; 3 Centre of Mental Health, University Health Network, 200 Elizabeth St, Toronto, ON M5G 2C4 Canada; 4 Temerty Faculty of Medicine, University of Toronto, Toronto, ON Canada

**Keywords:** Digital workflow, Patient care, COVID-19, Patient-centred, Psychosocial oncology

## Abstract

**Introduction:**

The COVID-19 pandemic created an urgent need for an innovative method of care delivery for psychosocial oncology. The psychosocial oncology services at the University Health Network in Toronto, Canada transitioned expeditiously to digital technologies that were readily available and accessible for patients and clinicians, facilitating care provision while reducing the transmission of COVID-19. This study aims to provide a validated framework for transitioning to digital delivery methods of care.

**Methods:**

A quality improvement team was established and tasked with successfully transitioning services from primarily in-person to digital delivery methods of care quickly and seamlessly. This included analyzing the psychosocial oncology workflow, planning and implementing a digital transition, and collecting data and feedback on the impact of this digital workflow through the use of surveys.

**Results:**

The average response rate of the surveys was 68.0%. Feedback and data collection demonstrated that more than 90% of psychosocial oncology processes were completed with digital tools following the transition with limited impact on clinical delivery. The clinicians reported feeling confident and satisfied providing care using digital workflow tools.

**Conclusion:**

The psychosocial oncology quality improvement team at the University Health Network provides a validated framework for transitioning to new methods of delivering care. As technology continues to develop, guidance on transitioning clinics and departments to new digital tools will be crucial for healthcare institutions. The framework provided in this study can be utilized to ensure the successful implementation of new technologies.

**Supplementary Information:**

The online version contains supplementary material available at 10.1186/s12913-025-13609-5.

## Introduction

With the rapid spread of the SARS-CoV-2 virus in early 2020 and the World Health Organization (WHO) declaration of the COVID-19 pandemic [1] in March 2020, there was urgent need for drastic changes to the delivery of care for psychosocial oncology (PSO) at the University Health Network (UHN) in Toronto, Canada. The PSO services provide care for mental health challenges, for patients 18 years of age and above, within Canada, undergoing any form of oncology treatment. To decrease transmission of COVID-19, provision of care was adapted to reduce person-to-person contact [2, 3]. New methods of delivering care to were required, facilitating reduction of in-person contact while aligning with local guidelines provided by the Ontario provincial government and the UHN Infection Prevention and Control (IPAC) group. However, the lessons learned in improving healthcare accessibility through digital tools during the COVID-19 pandemic remain relevant, especially given ongoing challenges related to resource constraints and staffing shortages in healthcare, particularly in psychological care [4].

When deciding on new methods of care, it was important to ensure that quality of care was upheld. In-person psychosocial care allows clinicians to pick up on non-verbal cues from patients including body movements, eye contact, intoxication, and hygiene that may provide clues in clinical assessment. Furthermore, in-person consultations help ensure privacy in a safe space for patients [5]. An effective way to deliver health care in these conditions includes shifting current workflow procedures to virtual and digital technologies, facilitating patient-centred care while minimizing the risk of COVID-19 exposure to both patients and clinicians [2, 6–8]. The flexibility of telehealth enhances accessibility for clinicians and patients when managing physical and psychological illnesses [3, 9–11].

Previous attempts to transition to virtual care have met challenges with adoption of digital tools, stemming from poor buy-in and inadequate support for staff [12]. To facilitate digital transformation in an expedited manner, it will be essential for PSO departments to invest in strategies that prioritize effective change management.

The goal of this study is to improve operational effectiveness by implementing existing digital solutions at UHN to improve clinical care accessibility for psychosocial oncology clinicians (psychiatry, psychology, social work) at Princess Margaret Cancer Centre, Toronto, Canada during the COVID-19 pandemic.

## Methods

*Quality Improvement Team* The authors established a Quality Improvement (QI) team in March 2020 with diverse stakeholders and leadership, including administrators, social workers, psychiatrists, and triage leadership. Our team met regularly in working groups to discuss evaluation and implementation to improve care accessibility during the pandemic. These 30-minute meetings occurred two to three times per week, in addition to impromptu emergency sessions. Each session included evaluations of ongoing processes, assessments of workflow at a broad level, and reviews of current challenges associated with specific platforms. After first-line leadership consensus on the implementation plan, the investigators would meet with department leads for feedback. The authors received Research Ethics Board (REB) exemption and QI approval (21–0272) from the QI Review Committee (QIRC) within UHN. All procedures were approved by and performed according to the guidelines of the QI Review Committee within UHN. All participants that provided feedback, first provided electronic written informed consent to participate in this study.

### Analyzing psychosocial oncology workflow

**Fig. 1:**
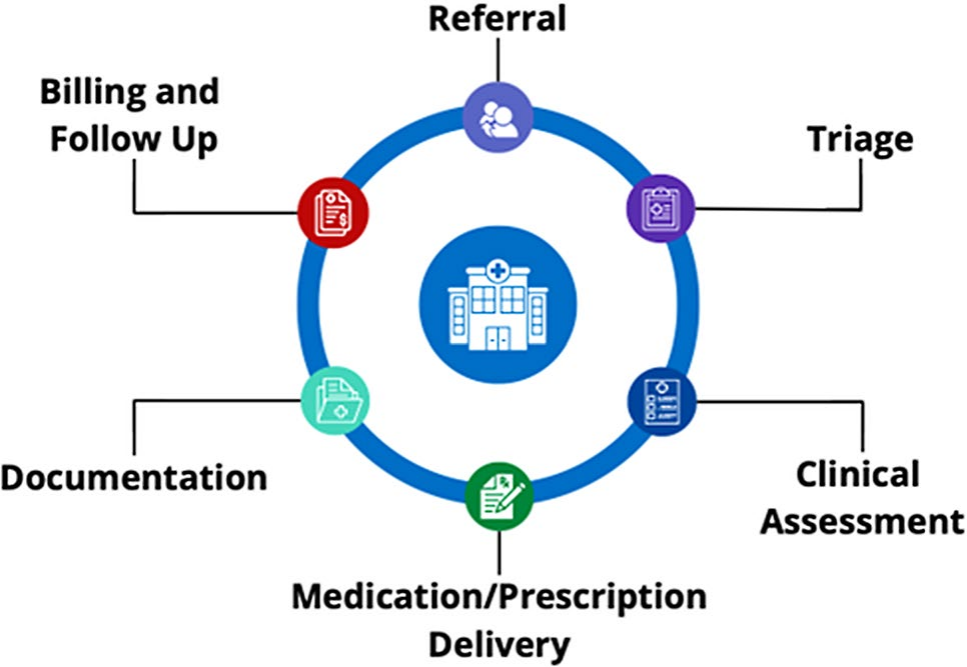
Psychosocial oncology workflow diagram

The investigators performed a comprehensive workflow analysis with the different stakeholders using the (Systems Engineering Initiative for Patient Safety) SEIPS 101 methodology to examine key aspects of the PSO workflow [13, 14]. As part of this analysis, the team developed a Journey Map to visualize and sequence major stages of the workflow, including referral, triage, clinical assessment, medication/prescription delivery, documentation, billing, and follow-up communication (Fig. 1).

### Planning and implementing digital transition

Digital technologies were reviewed from credible sources for practical accessibility and protection of health information. We reviewed the capabilities of digital technologies that were available and approved by UHN, as well as compliant with the Health Insurance Portability and Accountability Act (HIPAA), and the Personal Health Information Protection Act (PHIPA). All digital tools were cross-referenced with the national and provincial regulatory bodies including Canadian Medical Protective Association (CMPA) and Ontario Medical Association (OMA).

An assessment of the technologies that can be integrated into the current PSO workflow was completed. The internal assessment of technologies for integration into the PSO workflow was guided by national and provincial regulatory bodies including College of Physicians and Surgeons of Ontario (CPSO), CMPA. This process involved cross-referencing UHN-approved technologies, including Microsoft OneDrive, Microsoft Teams, Adobe PDF Editor, and newly implemented UHN processes, such as eFAX. Ad-hoc evaluations were performed to assess current technologies, their capabilities, and their alignment with PSO departmental needs. This functional analysis aimed to facilitate seamless workflow integration while supporting infection prevention and control protocols. After the QI team reviewed the assessment, the working group selected key quality indicators to measure changes between 2019 and 2021 (Table 1).

**Table 1:** Quality indicators for measuring change

Target	Indicator	Description	Data Source
Successful Transition to Digital Tools for Clinical Service Delivery	Percentage of clinical encounters conducted virtually	Measures the proportion of total clinical encounters completed via telehealth	Public Health Scheduling System (Appendix A)
	Average number of charting entries completed per week using Microsoft OneDrive	Assesses the transition from paper-based to digital charting.	Admin Survey (Appendix B)
	Average number of referrals processed per week using Microsoft Teams	Evaluates the efficiency of digital referral triaging.	Triage Survey (Appendix C)
	Average number of e-prescriptions transmitted directly from clinician to pharmacy using UHN’s electronic faxing system	Measures the utilization of digital prescription processing.	ePrescriptions Survey (Appendix D)
Ensure Continuity and Effectiveness of Care During Digital Transition	No-show assessment rates for new referrals and follow-ups	Percentage of scheduled new referral and follow-up appointments where patients did not attend.	Public Health Scheduling System (Appendix A)
	Average number of triage incidents	Count of unresolved issues or missed referrals during the triage process, indicating potential workflow gaps.	Triage Survey (Appendix C)
	Clinicians’ self-evaluated confidence in digital tools	Average confidence level reported by clinicians regarding their ability to effectively use individual digital tools for clinical tasks.	General Survey (Appendix E)
	Clinicians’ satisfaction with digital tools	Average satisfaction ratings for each type of digital tool, measured using a Likert scale.	General Survey (Appendix E)
	Clinicians’ satisfaction with digital training	Average satisfaction ratings for each type of digital tool training, measured using a Likert scale.	General Survey (Appendix E)
Improve Healthcare Accessibility	Wait times from referral to first appointment	Percentage of new patient referrals seen within two weeks.	Public Health Scheduling System (Appendix A)
	Percentage of patients outside of UHN’s catchment area	Percentage of patients whose postal codes fall outside of the defined UHN catchment area (Toronto, Canada), indicating potential shifts in patient demographics and accessibility.	Electronic Patient Records (Appendix F)

For this project, the definition of Telehealth appointment is using Ontario Telemedicine Network (OTN), Microsoft Teams (MST) and telephone platforms for patient encounters. Charting refers to the process of documenting a patient’s medical history, symptoms, diagnoses, treatments, and other relevant healthcare information in a medical record. Triage incidents are defined as unresolved issues or missed referrals. E-prescriptions are PDF prescriptions that were faxed by our organization’s electronic faxing system.

As organizational change can induce uncertainty, anxiety and stress for staff leading to burnout [15] and as healthcare providers were already experiencing burnout before the pandemic [16, 17], our goal was to minimize the stress created by implementing change. 1–2 different technologies were released every 4–6 weeks after smaller pilot testing with a smaller sample group [18]. To further assist with the transition, investigators prepared and disseminated educational materials on digital tools, offered individual support, provided on-demand support to the department and collected feedback from staff. Most importantly, investigators implemented phases to reduce the impact on clinic flow. Plan-Do-Study-Act (PDSA) cycles were used to assess the success of the digital transition [18].

### PDSA cycles for digital transition

The first PDSA cycle involved assessing the current PSO workflow to identify gaps in both paper-based and digital tools. A rapid workflow assessment was conducted by two clinical team members (CSQ, RS), followed by a review with clinical and administrative leadership. Using the SEIPS 101 journey map, we identified workflow areas that were misaligned with COVID-19 restrictions and determined digital solutions [13, 14]. The findings were presented to working groups, including administrative and psychiatry clinical teams, for validation. To ensure a smooth transition, a small-scale pilot was implemented with two clinicians who were early adopters of digital tools, minimizing disruption to the PSO team while assessing real-world feasibility. While real-time feedback was collected via group discussions, it was not formally recorded due to the lack of REB or QIRC approval. The process was iteratively adjusted based on this feedback, and workflow processes were refined by incorporating input from clinical, administrative, and technological stakeholders. This led to the identification of tools suitable for broader implementation in subsequent cycles.

In the second PDSA cycle, digital tools were launched solely for the psychiatry team, this team was selected based on clinician volume and the need for comprehensive care. Pilot programs were implemented for clinical digital assessments using OTN, documentation accessibility through OneDrive, and billing/follow-up communications through Outlook. Knowledge mobilization efforts included presentations to engage stakeholders and ensure proper tool usage. Working groups and one-on-one feedback sessions were conducted to assess technical knowledge gaps and usability challenges, though the findings were not formally recorded due to lack of REB and QIRC approval. Based on the feedback from clinical, administrative, and technology leaders, adjustments were made to optimize the implementation of the digital tools.

In the third and final PDSA cycle, all remaining digital tools were implemented across the program. We aimed to assess clinician confidence and satisfaction with the implemented technologies to ensure long-term adoption and effectiveness. Digital tools were implemented, including transitioning clinical assessments from OTN to MS Teams, integrating medication and prescription delivery using eFAX and Outlook, and implementing digital referral and triage workflows through Outlook and MS Teams. To support adoption, presentations, drop-in focus groups, and one-on-one training sessions were conducted to address technical concerns and enhance clinician proficiency. A structured evaluation was conducted using Jotform surveys with REB exemption and QIRC approval (21–0272) from our healthcare institution, to gather both quantitative and qualitative data on clinician satisfaction and tool effectiveness. Findings were analyzed and shared within the department, with data used to inform future refinements and enhance digital workflow adoption.

### Collection of feedback and data

The team established a data source and measurement method for each digital intervention. During the initial phase of transition, real-time feedback was collected via group discussions that aligned with the PDSA methodology. This included collecting data from national and provincial databases, including eCancercare, local organizational databases, such as Public Health Scheduling System (PHS) and Electronic Patient Records (EPR), departmental databases, such as outlook calendars, and the collection of staff feedback via surveys. All participants and stakeholders who provided feedback gave electronic written informed consent before completing any voluntary survey.

Following the implementation of the digital transition, a survey was completed by psychiatrists, psychologists, social workers, and administration, using an online HIPAA approved platform, JotForm [19]. Each of the four survey instruments were developed for this study to collect data on methods used to complete prescriptions, triage, charting and administrative processes, as well as staff experiences with the digital transition (Appendix G). These surveys were self-designed and were not piloted prior to implementation due to the impromptu nature of the project, which introduces certain limitations, including potential biases and challenges in ensuring reliability and validity. Data was collected in December 2021, following the acquisition of REB exemption and QIRC approval. Since the data was collected retroactively, categorical ranges were used in surveys instead of discrete data points to mitigate recall bias [20, 21]. As the surveys were administered at the end of 2021 and asked respondents to retrospectively reflect on practices in 2019, 2020, and 2021, they are best interpreted as providing descriptive, role-specific perspectives on practice change over time, rather than precise quantitative measures. Response rates varied across professional roles, with some groups overrepresented and others underrepresented (Appendix H), which may introduce bias and limit generalizability.

Several evidence-based strategies were implemented in this project to optimize stakeholder engagement. Stakeholder engagement provides the project leads with perspectives on topics that may be outside of the project lead or leads’ areas of expertise [22]. Also, higher survey response rates are important as surveys allow us to monitor progress and measure improvement [18]. To maximize response rates, the QI team sent out reminder emails for the surveys and personal emails to those who had not completed the survey.

### Analysis

All quantitative data from the databases were analyzed using descriptive statistics to assess trends over time. The data were used to calculate the percentage of interest for various metrics, which were then compared across three years to identify trends. For the percentage of virtual encounters, descriptive statistics were applied to determine whether the benchmark of 90% was achieved. Descriptive statistics were also used to analyze no-show assessment rates, wait times, and catchment area to observe any trends and differences between 2019, 2020, and 2021.

The quantitative analysis of the surveys was conducted using descriptive statistics and trend analysis. Response rates and completion rates were calculated for each individual survey, followed by the determination of average response and completion rates across all four surveys by computing the mean. A survey responder was defined as anyone who submitted a survey, regardless of completion, meaning partially completed surveys were included in the response rate calculation. Descriptive statistics were then employed to compute the average response for each question, enabling the observation of trends over time. Given that response rates were uneven across roles, findings may not fully represent all staff perspectives; nonetheless, the data provide descriptive insight into staff experiences of the digital transition.

Regarding qualitative data, real-time feedback was not recorded during the implementation of the digital tools, as the study was conducted in an impromptu manner without prior REB approval or QIRC approval. As a result, a full qualitative analysis of real-time feedback was not possible. While open-ended questions were included in the retroactive surveys, the feedback received in most surveys was not directly relevant to the digital tool implementation, except for the general survey (Appendix E). The feedback collected from the general survey was not addressed within this project as it was collected retroactively, therefore, it will not be analyzed in depth. However, an overview of the feedback will be provided by summarizing key quotes to provide insight on challenges and successes of the implementation.

## Results

The response rates to our surveys were collectively high. Across the four surveys (*n* = 4), the average completion rate was 79.9%, indicating that most respondents answered the majority of questions. The average response rate across our four surveys was 68.0%, with a total of 37 responses (Appendix H). This survey response rate was above the general benchmark of 35.7% for organizational surveys reported in the literature [23]. Clinician leave resulted in variations in our completion rates across the years.

### *Transition to* digital tools for clinical service delivery

**Fig. 2:**
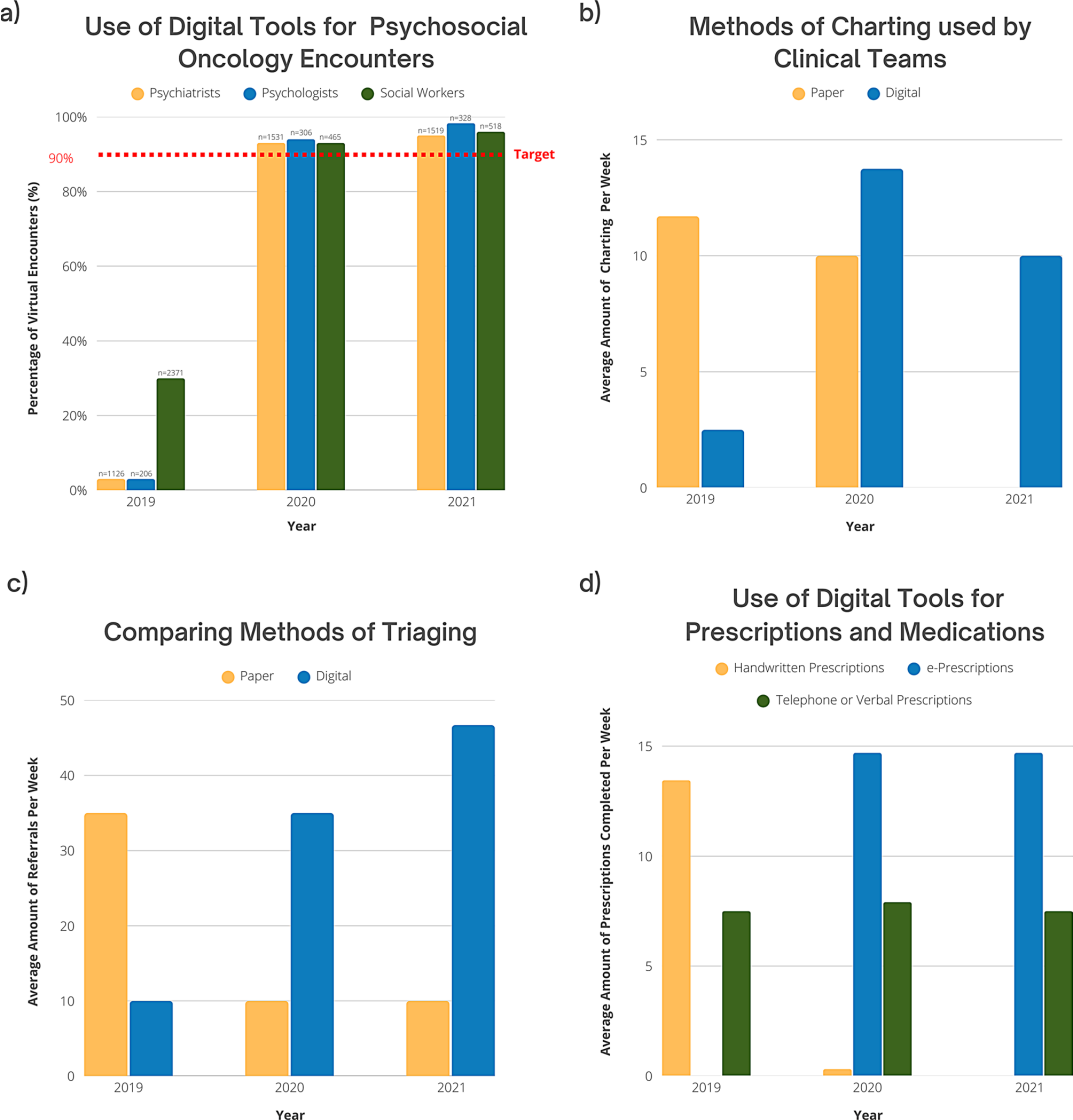
Measuring use of digital tools. This figure features four graphs that depict the use of digital tools by the PSO Department from 2019 to 2021: (**a**) Use of Digital Tools for Psychosocial Oncology Encounters; *n* indicates the number of patients with a virtual encounter per year (Appendix A), (**b**) Methods of Charting used by Clinical Teams (Appendix B), (**c**) Comparing Methods of Triaging (Appendix C), and (**d**) Use of Digital Tools for Prescriptions and Medications (Appendix D)

Following the implementation of digital tools, data and surveys were collected to evaluate the success of the implementation (Table 1). As shown in Fig. 2, in 2019, only 3% of psychiatry visits (*n* = 31), 3% of psychology visits (*n* = 6), and 30% of social worker encounters (*n* = 713) were virtual. By 2020, this increased substantially to 93% (*n* = 1384), 94% (*n* = 290), and 93% (*n* = 435), respectively. By 2021, virtual care accounted for 95% of psychiatry visits (*n* = 1384), 98% of psychology visits (*n* = 311), and 96% of social worker encounters (*n* = 485), surpassing our goal of converting 90% of in-person visits to virtual care. The notable decrease in total social work encounters from 2019 to 2020 and 2021 is attributable to changes in tracking systems and the concurrent use of two separate systems during this period, which has made complete data retrieval infeasible due to the time elapsed. Despite this limitation, the consistent increase in the proportion of patients receiving virtual care observed in both psychiatry and psychology, where data collection was stable and consistent, supports the reliability of our findings regarding the uptake of virtual care across disciplines.

A survey of the administration team (*n* = 3) (Appendix B) revealed a categorical shift from paper-based to digital charting in the PSO department. In 2019, the department primarily relied on paper records, with some administrators logging over 20 paper-based entries per week. By 2021, all charting had transitioned to digital, with no paper charting reported across administrative roles (Fig. 2). Additionally, while responses were limited, all administrators reported exclusively using digital tools for billing and follow-ups in 2020 and 2021, compared to 50% in 2019 (Appendix B).

Similarly, another survey (*n* = 6) (Appendix C) examined the transition from paper-based to digital triaging of new referrals. In 2019, administrators processed 40–59 paper referrals per week, psychiatrists handled 20–39, and social workers ranged from 0 to 19 to 40–59. By 2020, paper referrals had declined substantially across all roles, with most staff processing fewer than 20 per week. By 2021, digital triaging became the dominant method, with administrators processing 60–79 digital referrals per week, psychiatrists 20–39, and social workers fully transitioning to digital platforms (Fig. 2).

Lastly, a survey on prescription methods (*n* = 8) (Appendix D) compared the use of handwritten, telephone/verbal, and e-prescriptions. In 2019, all prescriptions were handwritten or verbal. By 2020, e-prescriptions accounted for 64.1% of all prescriptions, rising to 66.2% by 2021. Handwritten prescriptions declined from 100% in 2019 to just 1.4% in 2020 and were eliminated by 2021. Verbal prescriptions declined from 35.8% in 2019 to 33.8% in 2021, indicating a gradual but persistent shift toward digital prescribing (Fig. 2).

### Continuity and effectiveness of care during digital transition

**Fig. 3:**
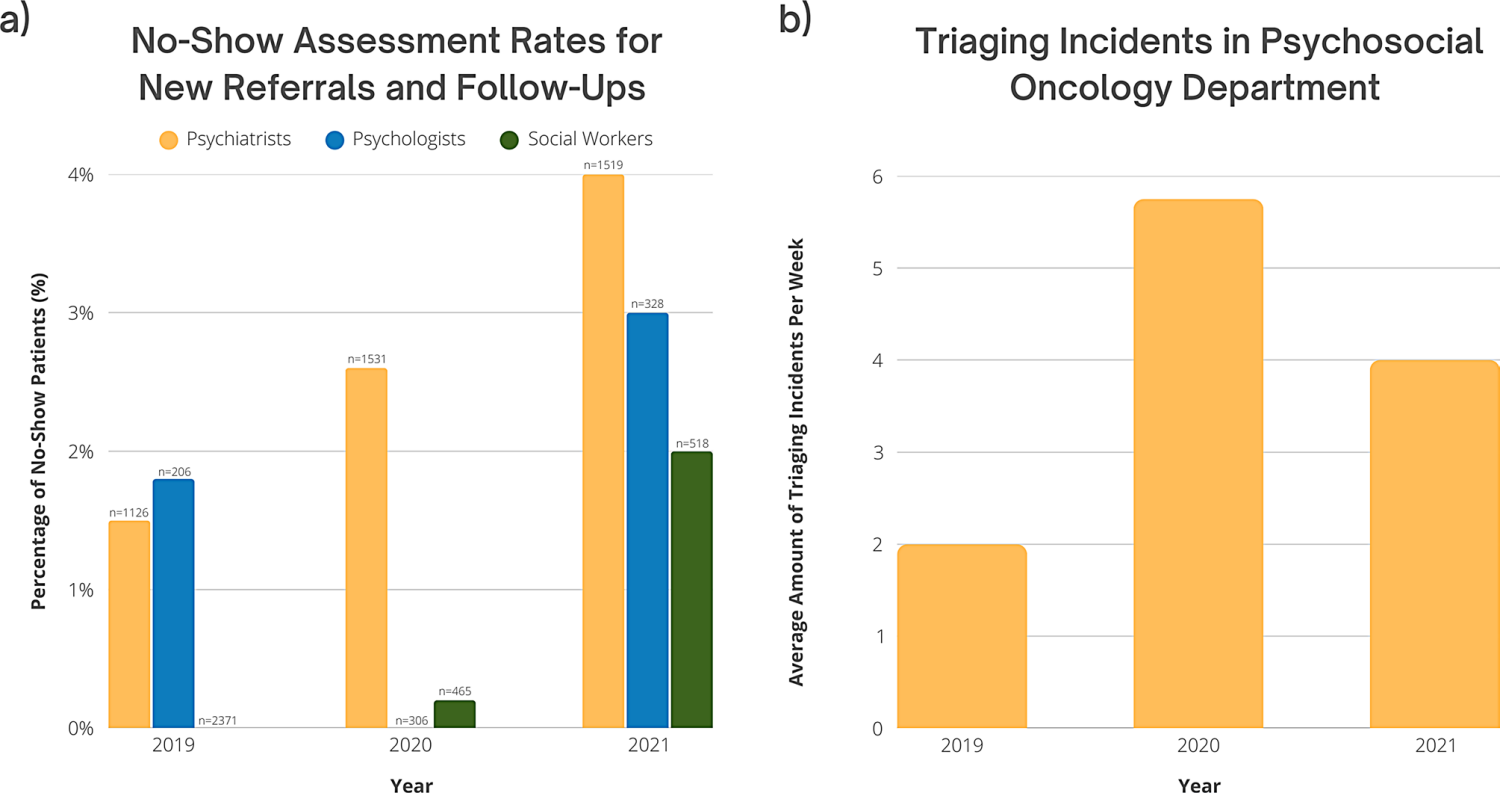
Assessing service provisions. Trends in service provision within the Department of PSO from 2019 to 2021 are presented in two panels: (**a**) No-show rates for new referrals and follow-up appointments, *n* indicates the number of patients who did not attend each year (Appendix A), and (**b**) Number of triaging incidents reported in the department (Appendix C)

The transition to digital tools was assessed by analyzing no-show assessment rates, incident triaging, PSO clinicians’ confidence with digital tools, and satisfaction with both the tools and training methods. Despite an increase in no-show assessment rates, the highest recorded rate was 4% in 2021 among psychiatry patients, indicating that service delivery remained largely uninterrupted. Triaging incidents also rose during this period, peaking in 2020, but these changes were not substantial enough to suggest significant disruptions in care. Overall, the data suggest that the digital transition had minimal impact on continuity and effectiveness of care, with no-show rates remaining low and service provision remaining feasible (Fig. 3).

**Fig. 4:**
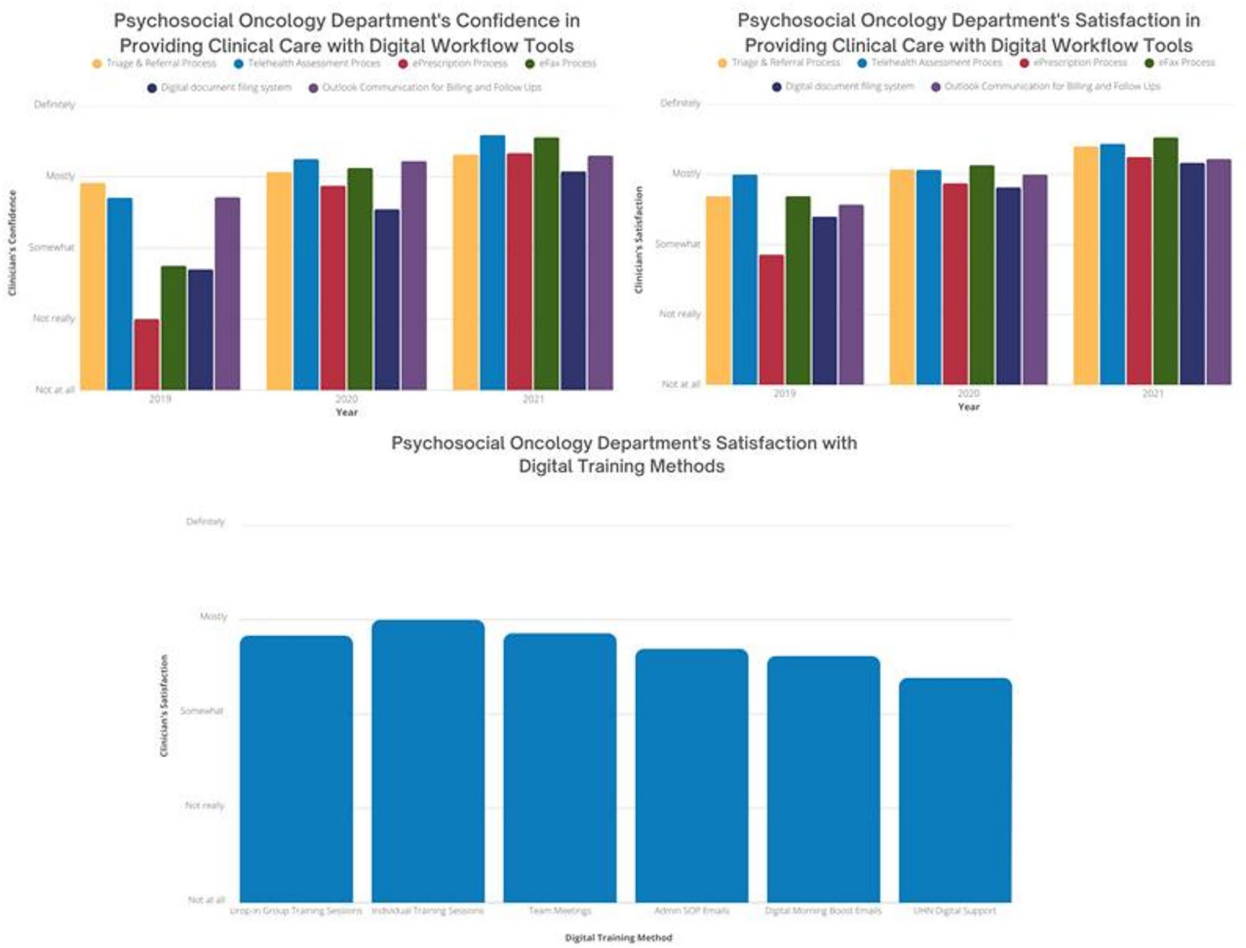
Psychosocial oncology providers’ experiences with digital transition. This figure features three graphs that depict PSO providers’ experiences with digital tools and training: (**a**) Psychosocial Oncology Department’s Confidence in Providing Clinical Care with Digital Workflow Tools (Appendix C), (**b**) Psychosocial Oncology Department’s Satisfaction in Providing Clinical Care with Digital Workflow Tools (Appendix C), and (**c**) Psychosocial Oncology Department’s Satisfaction with Digital Training Methods (Appendix C)

Surveying clinicians in the PSO department (*n* = 20) (Appendix C) yielded valuable department-wide insights. The average percentage of PSO department members who were mostly or definitely confident in using the 6 digital workflow tools increased significantly, from 53.4% in 2019 to 86.9% in 2021. Satisfaction with these 6 tools also rose from 46.5% in 2019 to 80.9% in 2021 (Fig. 4). Among the tools facilitating practice change, team meetings and group drop-in training sessions received the highest satisfaction ratings, with 66.7% (*n* = 12) and 71.4% (*n* = 14) of respondents indicating they were mostly or definitely satisfied with these resources, respectively. Additionally, on average, 59.9% of PSO department members expressed being mostly or definitely satisfied with all of the tools designed to facilitate practice change (Fig. 4).

### Improve healthcare accessibility

**Fig. 5:**
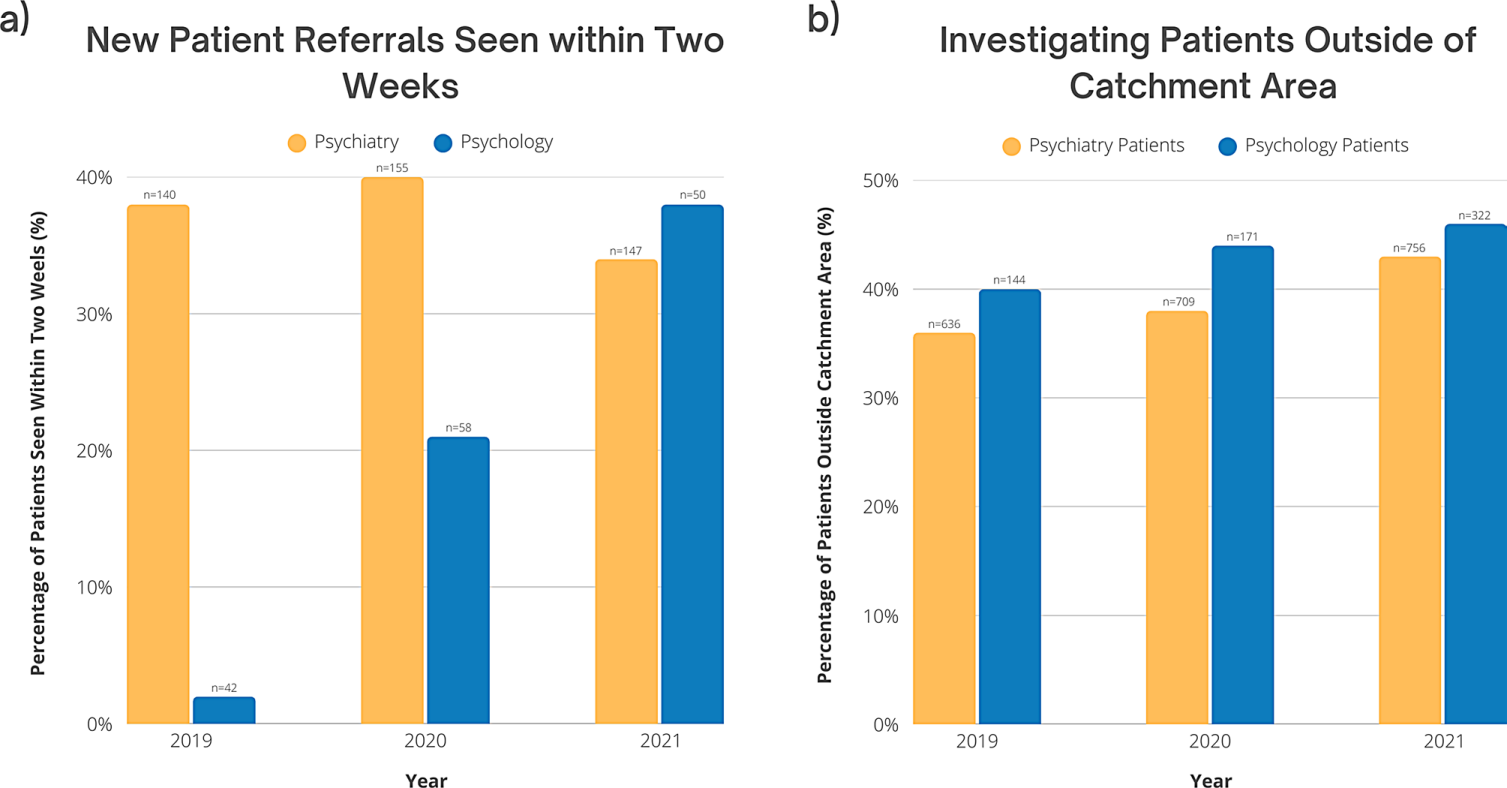
Investigating healthcare accessibility. This figure presents two graphs illustrating changes in healthcare accessibility in the PSO Department from 2019 to 2021 as a result of the implementation of digital tools: (**a**) New Patient Referrals Seen within Two Weeks, *n* indicates the number of patients who were seen within two weeks each year (Appendix A), and (**b**) Investigating Patients Outside of Catchment Area, *n* indicates the number of patients outside of catchment area who were seen each year (Appendix F). Please note that the total number of patients in graph (**b**) differs from other graphs due to the use of a different data source that included catchment area information

Wait times and patient catchment area were analyzed to assess whether digital tools improved healthcare accessibility. From 2019 to 2021, the proportion of psychiatry and psychology patients seen within two weeks increased from 20.1% (*n* = 54) to 36.0% (*n* = 69) (Appendix A), bringing the PSO department closer to aligning with Cancer Care Ontario’s guideline that patients with depression should be seen within this timeframe [24]. During the same period, the number of new patients rose from 182 in 2019 to 213 in 2020 and 197 in 2021, while the total number of patients increased from 1,332 in 2019 to 1,837 in 2020 and 1,847 in 2021 (Appendix A). Provider capacity also expanded, with psychology staffing increasing from 1.1 Full-Time Equivalent (FTE) in 2019 and 2020 to 1.7 FTE in 2021. and psychiatry increasing from 4.3 FTE in 2019 to 6.1 FTE in 2020 and 2021. Given that total patient volumes increased alongside staffing capacity, the observed improvement in timely access is unlikely to be solely attributable to increased staffing, suggesting that digital tools likely played an important role. Additionally, analysis of patient postal codes showed an increase in out-of-catchment patients from 38.4% (*n* = 290) in 2019 to 44.6% (*n* = 475) in 2021 (Fig. 5), indicating that digital care delivery expanded the department’s reach beyond the local service area. Please note that the total number of patients reported for catchment area analysis differs from other graphs due to the use of a separate data source, which accounts for variations in absolute figures.

## Discussion

### Summary of main findings

Our study tracked several quality indicators to characterize the effectiveness of the PSO department’s digital transition strategy. Underlying this transition was the urgent and necessary prioritization of safety through the reduction of person-to-person contact. To achieve this goal there was particular interest in tracking the adoption rates of digital technologies in existing workflows.

Between 2020 and 2021, investigators found that digital tools were used at a higher rate than paper modalities compared to pre-pandemic usage where paper-usage dominated most clinical workflows. Part of this increased adoption may be attributed to the concurrent cultural shift towards socially distanced interaction, which may have bolstered buy-in from PSO staff. However, group discussions with PSO clinicians also found that there was an overall positive reception to the newly introduced digital tools, which may have contributed to the sustained usage of digital tools over a 2-year period. Furthermore, there was a noticeable increase in satisfaction from 2019, prior to the implementation of training and support tools for clinicians. This suggests that the inclusion of educational tools and dedicated support were important factors in effective digital transition. The reduction in weekly triaging incidents between 2020 and 2021 may also suggest that staff’s proficiency with the digital tool improved over this period.

Along with adoption rates, it was paramount to ensure that the services offered by PSO were accessible to its patient population despite the drastic switch from in-person to virtual visits. The switch to digital platforms saw improvements to timeliness of new referrals, particularly for psychology services where more patients were seen within 2 weeks over the study period. This may have been due to the increased scheduling flexibility offered by our virtual platform, allowing patients to see clinicians more conveniently during busy hours of the day. Despite this, there was also a slight increase in no-show assessment rates across services.

A notable finding was the increasing proportion of patients seen from outside Toronto, Ontario, the PSO’s catchment region, over the study period. Virtual care may serve as an alternative for patients living in remote areas who were previously restricted by the time and cost burden of transportation. This benefit may also extend to patients whose medical conditions are an obstacle to seeking care in-person or whose caregivers have difficulty accommodating in-person visits. Many of PSO’s patients are immunocompromised due to their conditions and treatment regimens and may prefer virtual care to minimize pathogen transmission risk. These benefits of virtual care have the potential to extend beyond the pandemic to facilitate accessibility of PSO services.

Although qualitative feedback was not collected using a validated methodology, participants provided valuable insights that inform future directions. Key themes included challenges for elderly patients adapting to virtual care, concerns about the loss of nonverbal cues in virtual assessments, the need for automated appointment reminders and confirmation notifications, and frustrations with the burden of using multiple digital platforms. These observations will guide next steps in streamlining workflows and improving accessibility and user experience across digital tools.

### Implications for psychosocial oncology

Following the COVID-19 pandemic, the importance of crisis preparation has been impressed throughout healthcare. Our digital transition strategy provides Psychosocial Oncology departments with a framework that serves the dual purpose of crisis preparation and overall improvement to service accessibility. As a relatively young field, PSO should seek to improve the reach of its services as they become more integral in the care of patients living with life-altering diagnoses [25]. Based on our experience and the application of QI tools, we developed a practical, stepwise framework for implementing digital tools in psychosocial oncology settings (Table 2).

**Table 2:** Framework for implementing and transitioning to digital care

Step	Description	References
1. Identify and find the Problem	Determine the specific challenge in psychosocial oncology that digital tools can address, such as improving patient engagement, healthcare accessibility, privacy, security, or workflow efficiency.	[13, 14, 18, 26]
2. Assemble a QI Team	Form an interdisciplinary team, including clinicians, IT specialists, project managers, and researchers, to ensure diverse expertise and perspectives.	[14, 26–28]
3. Engage Stakeholders and Obtain Buy-in	Secure buy-in from department leadership, clinicians, and key stakeholders by aligning digital tools with organizational goals and patient needs.	[14, 29–31]
4. Establish Methods of Communication with Clinical Groups and Admins	Use formal and informal meetings, email, or approved communication platforms to collaborate with clinical groups and administrators. Gather insights on needs, challenges, and opportunities for digital tool integration while mapping workflow processes to identify optimal implementation points. Ensure communication mechanisms remain open throughout the project lifecycle.	[14, 32, 33]
5. Connect with Patients	Involve patients early to identify their needs and ensure digital tools align with their psychosocial support requirements. Conduct usability testing and collect feedback to prioritize ease of use, accessibility, and relevance to patient care.	[34–36]
6. Assess available and eligible digital tools	Conduct a scan of digital tools compliant with organizational policies. Assess tools based on feasibility, accessibility, security, and practicality. Evaluate implementation feasibility in terms of resources, time, and technical infrastructure.	[37, 38]
7. Develop an evaluation strategy	Clearly define project objectives and performance indicators to measure success. Establish evaluation criteria that assess implementation feasibility, accessibility, impact on clinical workflows, and effects on patient experience. Assign responsibility for monitoring and analysis to ensure accountability.	[39–41]
8. Develop and implement a rollout strategy	Design a phased implementation plan, including pilot testing, iterative refinements, and full-scale deployment. Provide staff training and technical support to ensure smooth adoption with minimal disruption to workflows.	[42, 43]
9. Knowledge Translation Internally	Educate internal teams on the benefits and functionality of digital tools through targeted training sessions, resource materials, and ongoing support. Ensure adoption strategies minimize workflow disruptions and foster sustained engagement.	[44–46]
10. Review Data and Improve Systems to Meet Needs	Continuously review user feedback from patients and staff to assess effectiveness. Identify challenges, refine functionalities, and implement iterative improvements based on real-world usage to optimize impact and sustainability. Establish a governance structure for ongoing evaluation and refinement.	[40, 47, 48]

Our strategy was designed to facilitate an expedited, yet controlled introduction of new digital workflows to our clinicians with a focus on effective change management. Our strategy utilized modern change management principles including iterative communication with leadership and stakeholders throughout the QI process, road mapping, dissemination of training materials, and on-demand support [49]. As a result, the obstacles faced were largely due to time constraints rather than pushback from stakeholders, who reported an overall positive reception. Positive outcomes were reflected by our quality indicators showing high usage of new digital solutions and improved service delivery, including accessibility.

With our digital transition in place, our department is now equipped to give patients the choice between in-person and virtual appointments, without impacting our clinician’s workflows. The shift to virtual care has continued to evolve, with increasing adoption and improvements in technology expanding its reach. Recent evidence suggests minimal differences between telehealth and in-person psychotherapy when caring for patients with physical conditions requiring psychological support [50]. This has allowed for a broader catchment area, enhancing accessibility for patients who may have faced barriers to in-person care. While we did not formally track post-pandemic outcomes, our digital transition framework aligns with these broader trends and has the potential to sustain benefits in process efficiency, service accessibility, and crisis preparedness.

### Challenges and limitations

There were several challenges faced during the digital transition process. The initial challenges included buy-in from leadership and stakeholders. There was an immense amount of change in work during and beyond the COVID-19 pandemic such as burnout and regular stress [15]. To sustain engagement with stakeholders, the QI team consistently met with program leadership to enhance bidirectional communication. As PSO sits within the larger UHN environment, investigators also needed to align with overall changes in digital processes within the organization. This required connecting with other working groups and balancing timelines and data transparency.

Additionally, the COVID-19 pandemic created an urgent need for adaptive changes to deliver psychosocial health care, necessitating an impromptu implementation that resulted in an ad-hoc analysis. Given the rapid nature of this transition, clear process metrics were not predefined, as REB exemption and QIRC approval to study the implementation were not received until 2021. As a result, investigators retrospectively tracked relevant indicators using available data sources, such as electronic medical records and databases, whenever possible. For certain processes, alternative metrics may have provided additional insights; for example, a throughput time analysis for referral triaging could offer a more precise measure of efficiency. These considerations will inform future PDSA iterations.

Moreover, some metrics were derived from retrospective surveys when objective data were unavailable, which introduced potential recall bias. Participants’ recollections may have been influenced by the time elapsed since implementation and external factors, such as workload or personal leave. The time gap between the implementation and survey responses is a critical factor, as recall bias tends to increase with the length of time between the event and the recollection [20]. This could lead to participants overestimating or underestimating their experiences, potentially distorting the reported outcomes. While trends in survey responses aligned with objective indicators, we acknowledge that recall bias may have contributed to variability in the data, limiting the accuracy of the results.

Furthermore, the surveys were self-designed and not piloted prior to implementation, which introduces additional limitations. The lack of piloting may have affected the reliability and validity of the data, as potential issues with question clarity, response interpretation, or measurement consistency were not identified in advance [51]. Additionally, self-designed surveys may introduce biases based on the perspectives of the study team, potentially influencing how questions were framed and interpreted [52]. Response rates varied across professional roles, with some groups overrepresented and others underrepresented, which may limit the generalizability of survey findings to the entire staff population. These limitations should be considered when interpreting the findings, and future research may benefit from pre-testing survey instruments to enhance their robustness.

Finally, another limitation of this study is the absence of follow-up data from subsequent years, which restricted our ability to assess the long-term sustainability and outcomes of the digital transition. While initial implementation data and short-term metrics were captured, ongoing evaluation is necessary to determine whether improvements in access and workflow efficiency were maintained over time. Incorporating longitudinal data in future quality improvement cycles would provide greater insight into program sustainability and evolving healthcare needs post-pandemic.

## Conclusion

This study aims to provide a novel resource for PSO departments looking to improve operational effectiveness by integrating digital solutions. This project began by recruiting stakeholders from leadership and different areas of the workflow to form an interdisciplinary team. Our team identified the workflow process involved in our department using QI-approved methodology.

After reviewing the current resources and strategic plan of our department, the authors provided and implemented a transition plan in PDSA segments. The authors outlined clear metrics and data collection tools for initial and ongoing data capture to evaluate the effectiveness of digital tools used and collect feedback from staff on the transition. Our approach emphasized the synergy of quantitative and qualitative metrics to appreciate all stakeholders’ experiences through the process. To support the transition, learning material and support tools were prepared to help with transitioning to the digital platform.

Our data showed high adoption of new digital solutions by our department following our change management strategy. The investigators also found that our services were delivered more efficiently with the improved ability to intake new referrals and increased reach of our services. Overall, our digital transition strategy proved to be an effective approach to facilitating change management, improving process efficiency, and making our services more accessibility in a time of crisis and beyond.

## Supplementary Information

Below is the link to the electronic supplementary material.

Supplementary Material 1

Supplementary Material 2

Supplementary Material 3

Supplementary Material 4

Supplementary Material 5

Supplementary Material 6

Supplementary Material 7

Supplementary Material 8

Supplementary Material 9

## Data Availability

The datasets have been included in the appendices of this manuscript.

## Abbreviations

CCO: Cancer Care Ontario
CMPA: Canadian Medical Protective Association
CPSO: College of Physicians and Surgeons of Ontario
EPR: Electronic Patient Records
FTE: Full-Time Equivalent
HIPAA: Health Insurance Portability and Accountability Act
IPAC: Infection Prevention and Control
MST: Microsoft Teams
OMA: Ontario Medical Association
OTN: Ontario Telemedicine Network
PDSA: Plan-Do-Study-Act
PHIPA: Personal Health Information Protection Act
PHS: Public Health Scheduling System
PSO: Psychosocial Oncology
QI: Quality Improvement;
QIRC: Quality Improvement Review Committee
SEIPS: Systems Engineering Initiative for Patient Safety
UHN: University Health Network
WHO: : World Health Organization
