# Supplementary material for: Quality improvement project to transition psychosocial oncology clinical care to a telehealth workflow during the COVID-19 pandemic: a quasi-experimental study
Source: BMC Health Serv Res. 2025 Nov 11;25:1460. doi: 10.1186/s12913-025-13609-5 (PMC12606822; doi:10.1186/s12913-025-13609-5)
Supplement: Supplementary file 1 — Supplementary Material 1 [file 12913_2025_13609_MOESM1_ESM.pdf]

In order to submit this form, you should open it with Adobe Acrobat Reader.

Survey 1

PSO Digital: Quality improvement

Survey: Admin Process

PSO is currently in the stages of reassessing digital integration and we are interested in your input to improve the accessibility and patient-centred care of our department.
Thank you for considering completing this HIPAA-compliant, secure survey for the digital integration within Psychosocial Oncology (PSO). If there are challenges with the format of their survey, please contact us to arrange alternate platforms (paper copy). This survey is optional.
This information will be stored on a secure, UHN approved, ONEDRIVE account and this information will be kept for 2 years. If you give us permission to contact you for further information to aid in quality improvement, please give us your contact information in the space given. This information will be seen by the QI PSO Digital team and kept for 2 years.

For the following questions, we will be asking information from many months ago, and we'd like you to take a moment to visualize the specific 2-month time period from different years. The time frame will be from September to October either from 2019, 2020 or more recently, 2021. This will help us with accurate information from these past time periods.

1) In the 2 month period from September 2019 to October 2019.

Before the COVID pandemic

How many PAPER CHARTS were used for all of your clinical team (psychiatrists, psychologist, social work, music therapy, etc) in 1 week?

- ◯ 0
- ◯ 1-5
- ◯ 6-10
- ◯ 11-15
- ◯ 16-20
- ◯ 20+

How many DIGITAL CHARTS (OneDrive / Sharepoint) were used for all of your clinical team (psychiatrists, psychologist, social work, music therapy, etc) in 1 week?

- ◯ 0
- ◯ 1-5
- ◯ 6-10
- ◯ 11-15
- ◯ 16-20
- ◯ 20+

How many of your psychiatrists were using the Outlook calendar for communication of follow-up & billing information?

[ ]

How many of your psychiatrists were NOT using the Outlook calendar for communication of follow-up & billing information?

[ ]

2) In the 2 month period from September 2020 to October 2020:

During the second wave of the COVID pandemic

How many PAPER CHARTS were used for all of your clinical team (psychiatrists, psychologist, social work, music therapy, etc) in 1 week?

- ◯ 0
- ◯ 1-5
- ◯ 6-10
- ◯ 11-15
- ◯ 16-20
- ◯ 20+

How many DIGITAL CHARTS (OneDrive / Sharepoint) were used for all of your clinical team (psychiatrists, psychologist, social work, music therapy, etc) in 1 week?

- ◯ 0
- ◯ 1-5
- ◯ 6-10
- ◯ 11-15
- ◯ 16-20
- ◯ 20+

How many of your psychiatrists were using the Outlook calendar for communication of follow-up & billing information?

[ ]

How many of your psychiatrists were NOT using the Outlook calendar for communication of follow-up & billing information?

[ ]

3) In the 2 month period from September 2021 to October 2021:

During the fourth wave of the COVID pandemic

How many PAPER CHARTS were used for all of your clinical team (psychiatrists, psychologist, social work, music therapy, etc) in 1 week? *

- ◯ 0
- ◯ 1-5
- ◯ 6-10
- ◯ 11-15
- ◯ 16-20
- ◯ 20+

How many DIGITAL CHARTS (OneDrive / Sharepoint) were used for all of your clinical team (psychiatrists, psychologist, social work, music therapy, etc) in 1 week? *

- ◯ 0
- ◯ 1-5
- ◯ 6-10
- ◯ 11-15
- ◯ 16-20
- ◯ 20+

How many of your psychiatrists were using the Outlook calendar for communication of follow-up & billing information?

How many of your psychiatrists were NOT using the Outlook calendar for communication of follow-up & billing information?

4) Additional comments or suggestions about the charting process?

Submit Form

In order to submit this form, you should open it with Adobe Acrobat Reader.

Survey 2

PSO Digital: Quality Improvement

Survey: ePrescription Process

PSO is currently in the stages of reassessing digital integration and we are interested in your input to improve the accessibility and patient-centred care of our department.
Thank you for considering completing this HIPAA-compliant, secure survey for the digital integration within Psychosocial Oncology (PSO). If there are challenges with the format of their survey, please contact us to arrange alternate platforms (paper copy). This survey is optional.
This information will be stored on a secure, UHN approved, ONEDRIVE account and this information will be kept for 2 years. If you give us permission to contact you for further information to aid in quality improvement, please give us your contact information in the space given. This information will be seen by the QI PSO Digital team and kept for 2 years.

1) What is your primary role in PSO?

Please select: *

For the following questions, we will be asking information from many months ago, and we'd like you to take a moment to visualize the specific 2-month time period from different years. The time frame will be from September to October either from 2019, 2020 or more recently, 2021. This will help us with accurate information from these past time periods.

2) From the period of September 2019 to October 2019, on average per psychiatrist:

Before the COVID pandemic

How many E-prescriptions (ELECTRONIC prescriptions) were completed? *

- ◯ 0
- ◯ 1-5
- ◯ 6-10
- ◯ 11-15
- ◯ 16-20
- ◯ 20+

How many HANDWRITTEN (ink) prescriptions were completed? *

- ◯ 0
- ◯ 1-5
- ◯ 6-10
- ◯ 11-15
- ◯ 16-20
- ◯ 20+

How many TELEPHONE or VERBAL prescriptions were completed? *

- ◯ 0
- ◯ 1-5
- ◯ 6-10
- ◯ 11-15
- ◯ 16-20
- ◯ 20+
- ◯ N/A

3) From the period of September 2020 to October 2020, on average per psychiatrist:

During the second wave of the COVID pandemic

How many E-prescriptions (ELECTRONIC prescriptions) were completed? *

- ◯ 0
- ◯ 1-5
- ◯ 6-10
- ◯ 11-15
- ◯ 16-20
- ◯ 20+

How many HANDWRITTEN (ink) prescriptions were completed? *

- ◯ 0
- ◯ 1-5
- ◯ 6-10
- ◯ 11-15
- ◯ 16-20
- ◯ 20+

How many TELEPHONE or VERBAL prescriptions were completed? *

- ◯ 0
- ◯ 1-5
- ◯ 6-10

◯ 11-15
◯ 16-20
◯ 20+
◯ N/A

4) From the period of September 2021 to October 2021, on average per psychiatrist:

During the fourth wave of the COVID pandemic

How many E-prescriptions (ELECTRONIC prescriptions) were completed? *

◯ 0
◯ 1-5
◯ 6-10
◯ 11-15
◯ 16-20
◯ 20+

How many HANDWRITTEN (ink) prescriptions were completed? *

◯ 0
◯ 1-5
◯ 6-10
◯ 11-15
◯ 16-20
◯ 20+

How many TELEPHONE or VERBAL prescriptions were completed? *

◯ 0
◯ 1-5
◯ 6-10
◯ 11-15
◯ 16-20
◯ 20+
◯ N/A

5) Additional comments or suggestions about the eprescription process?

Submit Form

In order to submit this form, you should open it with Adobe Acrobat Reader.

Survey 3

PSO Digital: Quality Improvement

The PSO team's perceptions on the digital transformation process

PSO is currently in the stages of reassessing digital integration and we are interested in your input to improve the accessibility and patient-centred care of our department.
Thank you for considering completing this HIPAA-compliant, secure survey for the digital integration within Psychosocial Oncology (PSO). If there are challenges with the format of their survey, please contact us to arrange alternate platforms (paper copy). This survey is optional.
This information will be stored on a secure, UHN approved, ONEDRIVE account and this information will be kept for 2 years. If you give us permission to contact you for further information to aid in quality improvement, please give us your contact information in the space given. This information will be seen by the QI PSO Digital team and kept for 2 years.

1) What is your primary role in PSO?

Please select: *

Please explain your role/title

For the following questions, we will be asking information from many months ago, and we'd like you to take a moment to visualize the specific 2-month time period from different years. The time frame will be from September to October either from 2019, 2020 or more recently, 2021. This will help us with accurate information from these past time periods.

2) From the period of September 2019 to October 2019: How confident did you feel in providing clinical care with each digital workflow tool?

September 2019-October 2019: Before the COVID pandemic

	Not at all	Not really	Somewhat	Mostly	Definitely	N/A
Triage & Referral process	◯	◯	◯	◯	◯	◯
Telehealth assessment process (OTN/MS Teams/ Telephone)	◯	◯	◯	◯	◯	◯
ePrescription process	◯	◯	◯	◯	◯	◯
eFAX process	◯	◯	◯	◯	◯	◯
Digital document filing system	◯	◯	◯	◯	◯	◯
Outlook communication for billing and follow-ups	◯	◯	◯	◯	◯	◯

Which digital clinical care tool did you feel most confident with?

◯ Triage & Referral
◯ Telehealth assessment tools
◯ ePrescriptions
◯ eFAX
◯ Digital document filing
◯ Outlook communication for billing and follow-ups

Which digital clinical care tool did you feel least confident with?

◯ Triage & Referral
◯ Telehealth assessment tools
◯ ePrescriptions
◯ eFAX
◯ Digital document filing
◯ Outlook communication for billing and follow-ups

3) From the period of September 2020 to October 2020: How confident did you feel in providing clinical care with each digital workflow tool?

September 2020-October 2020: During the second phase of the COVID pandemic

	Not at all	Not really	Somewhat	Mostly	Definitely	N/A
Triage & Referral process	◯	◯	◯	◯	◯	◯

**Telehealth assessment process (OTN/MS Teams/ Telephone)**	◯	◯	◯	◯	◯	◯
**ePrescription process**	◯	◯	◯	◯	◯	◯
**eFAX process**	◯	◯	◯	◯	◯	◯
**Digital document filing system**	◯	◯	◯	◯	◯	◯
**Outlook communication for billing and follow-ups**	◯	◯	◯	◯	◯	◯

Which digital clinical care tool did you feel most confident with?

◯ Triage & Referral
◯ Telehealth assessment tools
◯ ePrescriptions
◯ eFAX
◯ Digital document filing
◯ Outlook communication for billing and follow-ups

Which digital clinical care tool did you feel least confident with?

◯ Triage & Referral
◯ Telehealth assessment tools
◯ ePrescriptions
◯ eFAX
◯ Digital document filing
◯ Outlook communication for billing and follow-ups

4) From the period of September 2021 to October 2021: How confident did you feel in providing clinical care with each digital workflow tool?

September 2021-October 2021: During the fourth phase of the COVID pandemic

	Not at all	Not really	Somewhat	Mostly	Definitely	N/A
**Triage & Referral process**	◯	◯	◯	◯	◯	◯
**Telehealth assessment process (OTN/MS Teams/ Telephone)**	◯	◯	◯	◯	◯	◯
**ePrescription process**	◯	◯	◯	◯	◯	◯
**eFAX process**	◯	◯	◯	◯	◯	◯

Digital document filing system	◯	◯	◯	◯	◯	◯
Outlook communication for billing and follow-ups	◯	◯	◯	◯	◯	◯

Which digital clinical care tool did you feel most confident with?

◯ Triage & Referral
◯ Telehealth assessment tools
◯ ePrescriptions
◯ eFAX
◯ Digital document filing
◯ Outlook communication for billing and follow-ups

Which digital clinical care tool did you feel least confident with?

◯ Triage & Referral
◯ Telehealth assessment tools
◯ ePrescriptions
◯ eFAX
◯ Digital document filing
◯ Outlook communication for billing and follow-ups

5) During the PSO Digital training phase in 2019, which type of support did you find helpful in building your confidence with these digital tools?

2019: During the First and Second phase of the COVID pandemic

	Not at all	Not really	Somewhat	Mostly	Definitely	N/A
Scheduled group drop-in training sessions	◯	◯	◯	◯	◯	◯
Scheduled individual training sessions	◯	◯	◯	◯	◯	◯
Team meetings	◯	◯	◯	◯	◯	◯
Admin SOP emails	◯	◯	◯	◯	◯	◯
Digital PSO morning boost emails	◯	◯	◯	◯	◯	◯
UHN digital support	◯	◯	◯	◯	◯	◯

Are there other strategies/resources that you think would be helpful to build confidence?

6) From the period of September 2019 to October 2019: How satisfied do you feel in providing clinical care with each digital workflow tool?

September 2019-October 2019: Before the COVID pandemic

	Not at all	Not really	Somewhat	Mostly	Definitely	N/A
Triage & Referral process	◯	◯	◯	◯	◯	◯
Telehealth assessment process (OTN/MS Teams/ Telephone)	◯	◯	◯	◯	◯	◯
ePrescription process	◯	◯	◯	◯	◯	◯
eFAX process	◯	◯	◯	◯	◯	◯
Digital document filing system	◯	◯	◯	◯	◯	◯
Outlook communication for billing and follow-ups	◯	◯	◯	◯	◯	◯

Which digital clinical care tool did you feel most satisfied with?

◯ Triage & Referral
◯ Telehealth assessment tools
◯ ePrescriptions
◯ eFAX
◯ Digital document filing
◯ Outlook communication for billing and follow-ups

Which digital clinical care tool did you feel least satisfied with?

◯ Triage & Referral
◯ Telehealth assessment tools
◯ ePrescriptions
◯ eFAX
◯ Digital document filing
◯ Outlook communication for billing and follow-ups

7) From the period of September 2020 to October 2020: How satisfied do you feel in providing clinical care with each digital workflow tool?

September 2020-October 2020: During the second wave of the COVID pandemic

	Not at all	Not really	Somewhat	Mostly	Definitely	N/A
**Triage & Referral process**	◯	◯	◯	◯	◯	◯
**Telehealth assessment process (OTN/MS Teams/ Telephone)**	◯	◯	◯	◯	◯	◯
**ePrescription process**	◯	◯	◯	◯	◯	◯
**eFAX process**	◯	◯	◯	◯	◯	◯
**Digital document filing system**	◯	◯	◯	◯	◯	◯
**Outlook communication for billing and follow-ups**	◯	◯	◯	◯	◯	◯

Which digital clinical care tool did you feel most satisfied with?

◯ Triage & Referral
◯ Telehealth assessment tools
◯ ePrescriptions
◯ eFAX
◯ Digital document filing
◯ Outlook communication for billing and follow-ups

Which digital clinical care tool did you feel least satisfied with?

◯ Triage & Referral
◯ Telehealth assessment tools
◯ ePrescriptions
◯ eFAX
◯ Digital document filing
◯ Outlook communication for billing and follow-ups

8) From the period of September 2021 to October 2021: How satisfied do you feel in providing clinical care with each digital workflow tool?

September 2021-October 2021: During the fourth wave of the COVID pandemic

	Not at all	Not really	Somewhat	Mostly	Definitely	N/A
Triage & Referral process	◯	◯	◯	◯	◯	◯
Telehealth assessment process (OTN/MS Teams/ Telephone)	◯	◯	◯	◯	◯	◯
ePrescription process	◯	◯	◯	◯	◯	◯
eFAX process	◯	◯	◯	◯	◯	◯
Digital document filing system	◯	◯	◯	◯	◯	◯
Outlook communication for billing and follow-ups	◯	◯	◯	◯	◯	◯

Which digital clinical care tool did you feel most satisfied with?

◯ Triage & Referral
◯ Telehealth assessment tools
◯ ePrescriptions
◯ eFAX
◯ Digital document filing
◯ Outlook communication for billing and follow-ups

Which digital clinical care tool did you feel least satisfied with?

◯ Triage & Referral
◯ Telehealth assessment tools
◯ ePrescriptions
◯ eFAX
◯ Digital document filing
◯ Outlook communication for billing and follow-ups

9) How do you feel the digital tools have impacted patient care in 2021, in each category?

	Improved patient care	Similar patient care	Deteriorated patient care
Appointment attendance	◯	◯	◯

Accessibility for special populations (geriatric, palliative, lower SES, outside of catchment area, etc) ◯ ◯ ◯

Are there other areas of positive or negative impact on patient care?

10) Which tool(s) do you feel requires improvement?

Triage & Referral process

◯ Yes
◯ No

If yes, how could we improve it?

Telehealth clinical assessment tool - OTN/MS Teams/Telephone

◯ Yes
◯ No

If yes, how could we improve it?

ePrescriptions process

◯ Yes
◯ No

If yes, how could we improve it?

eFax process

◯ Yes
◯ No

If yes, how could we improve it?

Digital documentation filing

◯ Yes
◯ No

If yes, how could we improve it?

Outlook communication for billing & follow-ups

◯ Yes
◯ No

If yes, how could we improve it?

11) Additional comments or suggestions on the digital tools?

Submit Form

In order to submit this form, you should open it with Adobe Acrobat Reader.

Survey 4

PSO Digital: Quality Improvement

Survey: Triage Process

PSO is currently in the stages of reassessing digital integration and we are interested in your input to improve the accessibility and patient-centred care of our department.
Thank you for considering completing this HIPAA-compliant, secure survey for the digital integration within Psychosocial Oncology (PSO). If there are challenges with the format of their survey, please contact us to arrange alternate platforms (paper copy). This survey is optional.
This information will be stored on a secure, UHN approved, ONEDRIVE account and this information will be kept for 2 years. If you give us permission to contact you for further information to aid in quality improvement, please give us your contact information in the space given. This information will be seen by the QI PSO Digital team and kept for 2 years.

1) What is your primary role in PSO?

Please Select *

- ☐ Administration
- ☐ Psychology
- ☐ Psychiatry
- ☐ Social Work
- ☐ Other ____________

For the following questions, we will be asking information from many months ago, and we'd like you to take a moment to visualize the specific 2-month time period from different years. The time frame will be from September to October either from 2019, 2020 or more recently, 2021. This will help us with accurate information from these past time periods.

2) From the period of September 2019 to October 2019:

Before the COVID pandemic

How many new referrals went through the old, PAPER process in 1 week?

- ◯ 0-19
- ◯ 20-39
- ◯ 40-59

- 60-79
- 80-99
- 100-119
- 120+

How many new referrals went through the new, DIGITAL (MSTeams) process in 1 week?

- 0-19
- 20-39
- 40-59
- 60-79
- 80-99
- 100-119
- 120+

How many triage errors occurred during this time period (Sept 2019-Oct 2019)

- 0-4
- 5-9
- 10-14
- 15-19
- 20-24
- 25-29
- 30+

3) From the period of September 2020 to October 2020:

During the second wave of the COVID Pandemic

How many new referrals went through the old, PAPER process in 1 week?

- 0-19
- 20-39
- 40-59
- 60-79
- 80-99
- 100-119
- 120+

How many new referrals went through the new, DIGITAL (MSTeams) process in 1 week?

- 0-19
- 20-39
- 40-59
- 60-79
- 80-99

100-

- 120+

How many triage errors occurred during this time period (Sept 2019-Oct 2019)

- 0-4
- 5-9
- 10-14
- 15-19
- 20-24
- 25-29
- 30+

4) From the period of September 2021 to October 2021:

During the fourth wave of the COVID Pandemic

How many new referrals went through the old, PAPER process in 1 week?

- 0-19
- 20-39
- 40-59
- 60-79
- 80-99
- 100-119
- 120+

How many new referrals went through the new, DIGITAL (MSTeams) process in 1 week?

- 0-19
- 20-39
- 40-59
- 60-79
- 80-99
- 100-119
- 120+

How many triage errors occurred during this time period (Sept 2019-Oct 2019)

- 0-4
- 5-9
- 10-14
- 15-19
- 20-24
- 25-29
- 30+

5) With the older, Paper triage process:

How long did you feel the process took to complete one consult? How long would it take 1 consult, once received, to be booked from start to finish?

- ◯ 1-12hr
- ◯ 12-24hr
- ◯ 24-48hr
- ◯ 48-72hr
- ◯ 72hr or more

6) With the new, Digital (MS Teams) triage process:

How long did you feel the process took to complete one consult? How long would it take 1 consult, once received, to be booked from start to finish?

- ◯ 1-12hr
- ◯ 12-24hr
- ◯ 24-48hr
- ◯ 48-72hr
- ◯ 72hr or more

With respect to time, do you feel your part of the triage process is:

- ◯ Longer compared to the paper triage process
- ◯ Same amount of time compared to the paper triage process
- ◯ Shorter compared to the paper triage process
- ◯ Not sure

7) Additional comments or suggestions on the triage process?

Submit Form
